# Supplementary material for: Dysfunctional neuroplasticity in newly arrived Middle Eastern refugees in the U.S.: Association with environmental exposures and mental health symptoms
Source: PLoS One. 2020 Mar 6;15(3):e0230030. doi: 10.1371/journal.pone.0230030 (PMC7059916; doi:10.1371/journal.pone.0230030)
Supplement: Attachment — Submitted filename: Response to Reviewers.docx [file pone.0230030.s002.docx]

**Response to Reviewers (responses in bold)**

Journal requirements:

When submitting your revision, we need you to address these additional requirements.

1. Please ensure that your manuscript meets PLOS ONE's style requirements, including those for file naming. The PLOS ONE style templates can be found at
<http://www.journals.plos.org/plosone/s/file?id=wjVg/PLOSOne_formatting_sample_main_body.pdf> and <http://www.journals.plos.org/plosone/s/file?id=ba62/PLOSOne_formatting_sample_title_authors_affiliations.pdf>

**Thank you for alerting us to this requirement. We have now reviewed the template on the journal website and the manuscript has been formatted to meet the journals’ style requirements.**

2. We note that you have indicated that data from this study are available upon request. PLOS only allows data to be available upon request if there are legal or ethical restrictions on sharing data publicly. For information on unacceptable data access restrictions, please see <http://journals.plos.org/plosone/s/data-availability#loc-unacceptable-data-access-restrictions>.

In your revised cover letter, please address the following prompts:

a) If there are ethical or legal restrictions on sharing a de-identified data set, please explain them in detail (e.g., data contain potentially identifying or sensitive patient information) and who has imposed them (e.g., an ethics committee). Please also provide contact information for a data access committee, ethics committee, or other institutional body to which data requests may be sent.

b) If there are no restrictions, please upload the minimal anonymized data set necessary to replicate your study findings as either Supporting Information files or to a stable, public repository and provide us with the relevant URLs, DOIs, or accession numbers. Please see <http://www.bmj.com/content/340/bmj.c181.long> for guidelines on how to de-identify and prepare clinical data for publication. For a list of acceptable repositories, please see <http://journals.plos.org/plosone/s/data-availability#loc-recommended-repositories>.

We will update your Data Availability statement on your behalf to reflect the information you provide.

**We will be uploading the de-identified dataset as supporting information files.**

[Note: HTML markup is below. Please do not edit.]

Reviewers' comments:

Reviewer's Responses to Questions

**Comments to the Author**

1. Is the manuscript technically sound, and do the data support the conclusions?

The manuscript must describe a technically sound piece of scientific research with data that supports the conclusions. Experiments must have been conducted rigorously, with appropriate controls, replication, and sample sizes. The conclusions must be drawn appropriately based on the data presented.

Reviewer #1: Yes

Reviewer #2: Yes

2. Has the statistical analysis been performed appropriately and rigorously?

Reviewer #1: Yes

Reviewer #2: Yes

3. Have the authors made all data underlying the findings in their manuscript fully available?

The [PLOS Data policy](https://urldefense.com/v3/__http:/www.plosone.org/static/policies.action*sharing__;Iw!!HXCxUKc!ja5f0bXtI0e0Fq_FzvoARvK9PB4A76Mg2cMpGOThNnoBABZNPR1YOGBdadeYEvk$) requires authors to make all data underlying the findings described in their manuscript fully available without restriction, with rare exception (please refer to the Data Availability Statement in the manuscript PDF file). The data should be provided as part of the manuscript or its supporting information, or deposited to a public repository. For example, in addition to summary statistics, the data points behind means, medians and variance measures should be available. If there are restrictions on publicly sharing data—e.g. participant privacy or use of data from a third party—those must be specified.

Reviewer #1: Yes

Reviewer #2: Yes

4. Is the manuscript presented in an intelligible fashion and written in standard English?

PLOS ONE does not copyedit accepted manuscripts, so the language in submitted articles must be clear, correct, and unambiguous. Any typographical or grammatical errors should be corrected at revision, so please note any specific errors here.

Reviewer #1: Yes

Reviewer #2: Yes

5. Review Comments to the Author

Please use the space provided to explain your answers to the questions above. You may also include additional comments for the author, including concerns about dual publication, research ethics, or publication ethics. (Please upload your review as an attachment if it exceeds 20,000 characters)

Reviewer #1: The manuscript focused an important issue, and it was well written. It will contribute much for mental health fields. The methodological and statistical is appropriate. The interpretation of the results is rational. I think it should be accepted in the present form.

**We would like to thank the reviewer for reviewing the manuscript.**

Reviewer #2: This is an interesting paper where the authors have studied 64 newly arrived refugees from Syria and Iran living in Michigan USA. They were studied one month after their arrival by making an assessment on PTSD, depression, anxiety, self-reported mental health. neurotrophins- BDNF and NGF as proxy measures of neuroplasticity and exposure to heavy metals. The authors found independent associations between environmental exposures and neuroplasticity with different mental health problems. Few of the findings can be interpreted as different from what one expects.
As the authors argue this is a pioneer study where they have included two important measures, namely exposure to environmental toxins and neurobiological markers for measuring neuroplasticity in addition to classical descriptive studies in refugee trauma. This has certainly given an extra strength to this paper which will ultimately help us to understand refugee trauma mechanism better.

Abstract is well written and well presented. However, I wonder if the authors could avoid using the terminologies like predictor or predicted (e.g. line 49 ) as this is not a longitudinal study and the direction of causality is thus not very obvious. I would suggest the authors to be a little bit careful when they speculate for their findings. In line 64-66, they argue for role of neutrophins as facilitator of apoptosis which is far away from existing knowledge and understanding of its roles.
Introduction. Well written with relevant literatures
Methods: Sample selection is somewhat limited and the sample size is also rather small. Choice of the instruments and statistical methods are quite well explained and good.
Results are presented well both in tables and texts. I am just curious to know if the authors have sometimes thought about using neuroplasticity as a dependent variable in their analyses. It could have been interesting to look that too.
Discussions are also well balanced and presented. However, I think that authors are quite optimistic for using sophisticated biological parameters is studying refugee trauma which is not always possible specially in low-and middle- income countries.

**We would like to thank the reviewer for these valuable suggestions. We agree with the reviewer and have changed the terminology to make it clear that this is a cross-sectional study that cannot prove causality and therefore we have removed terms such as predictive in the Abstract and elsewhere.**

**As per reviewer’s suggestion, we have now deleted the sentence in line 64-66 of the original Abstract that mentioned role of neutrophins as facilitator of apoptosis.**

**Since we had hypothesized neuroplasticity to be potential biomarkers for the development of mental health disorders (PTSD, depression and anxiety), we had used it as an independent variable. However, the reviewer raised an interesting point regarding using neuroplasticity as a dependent variable. We ran the requested analysis, revised the text and added a Table 4. Interestingly, in separate linear regressions, the three mental health disorders were each associated with neuroplasticity. However, the models explained substantially lesser variance compared to when they were run as per our proposed model. We thank the reviewer for suggesting this alternative causative model.**

**We agree with the reviewer that the analysis of these biomarkers may not be possible in low-and middle-income countries due to the financial limitations. We have now acknowledged this as a potential limitation. However, we also believe that if our findings are confirmed in future studies, the costs for these analyses might be justified since measuring these biomarkers allows refugee mental health professionals to focus sparse resources on those with the largest needs.**

6. PLOS authors have the option to publish the peer review history of their article ([what does this mean?](https://urldefense.com/v3/__https:/journals.plos.org/plosone/s/editorial-and-peer-review-process*loc-peer-review-history__;Iw!!HXCxUKc!ja5f0bXtI0e0Fq_FzvoARvK9PB4A76Mg2cMpGOThNnoBABZNPR1YOGBdwNg7CSU$)). If published, this will include your full peer review and any attached files.


If you choose “no”, your identity will remain anonymous but your review may still be made public.


**Do you want your identity to be public for this peer review?** For information about this choice, including consent withdrawal, please see our [Privacy Policy](https://urldefense.com/v3/__https:/www.plos.org/privacy-policy__;!!HXCxUKc!ja5f0bXtI0e0Fq_FzvoARvK9PB4A76Mg2cMpGOThNnoBABZNPR1YOGBdxGon-rw$).

Reviewer #1: No

Reviewer #2: Yes: Suraj Bahadur Thapa


[NOTE: If reviewer comments were submitted as an attachment file, they will be attached to this email and accessible via the submission site. Please log into your account, locate the manuscript record, and check for the action link "View Attachments". If this link does not appear, there are no attachment files to be viewed.]


While revising your submission, please upload your figure files to the Preflight Analysis and Conversion Engine (PACE) digital diagnostic tool, [https://pacev2.apexcovantage.com/](https://urldefense.com/v3/__https:/pacev2.apexcovantage.com/__;!!HXCxUKc!ja5f0bXtI0e0Fq_FzvoARvK9PB4A76Mg2cMpGOThNnoBABZNPR1YOGBdyHTuYYo$). PACE helps ensure that figures meet PLOS requirements. To use PACE, you must first register as a user. Registration is free. Then, login and navigate to the UPLOAD tab, where you will find detailed instructions on how to use the tool. If you encounter any issues or have any questions when using PACE, please email us at [figures@plos.org](mailto:figures@plos.org). Please note that Supporting Information files do not need this step.
